# Clinical management of women with metastatic breast cancer: a descriptive study according to age group

**DOI:** 10.1186/1471-2407-6-179

**Published:** 2006-07-06

**Authors:** Klaartje Manders, Lonneke V van de Poll-Franse, Geert-Jan Creemers, Gerard Vreugdenhil, Maurice JC van der Sangen, Grard AP Nieuwenhuijzen, Rudi MH Roumen, Adri C Voogd

**Affiliations:** 1 Faculty of Medicine, Maastricht University, Maastricht, The Netherlands; 2 Comprehensive Cancer Center South, Eindhoven Cancer Registry, Eindhoven, The Netherlands; 3 Department of Internal Medicine, Catharina Hospital, Eindhoven, The Netherlands; 4 Department of Internal Medicine, Máxima Medical Center, Veldhoven, The Netherlands; 5 Department of Radiotherapy, Catharina Hospital, Eindhoven, The Netherlands; 6 Department of Surgery, Catharina Hospital, Eindhoven, The Netherlands; 7 Department of Surgery, Máxima Medical Center, Veldhoven, The Netherlands; 8 Department of Epidemiology, Maastricht University, PO Box 616, 6200 MD, The Netherlands

## Abstract

**Background:**

The primary aim of treatment of a patient who has developed metastatic disease is palliation. The objectives of the current study are to describe and quantify the clinical management of women with metastatic breast cancer from the diagnosis of metastatic disease until death and to analyze differences between age groups.

**Methods:**

Data were collected from the medical files of all patients (n = 116) who had died after December 31, 1999, after a diagnosis of metastatic breast cancer in two teaching hospitals in the south of the Netherlands.

**Results:**

Of the 116 patients included in our study, 10 (9%) already had metastatic disease at diagnosis and 106 developed distant disease after the diagnosis of localized breast cancer. Before they died, 70% of the 116 patients developed metastases in one or more bones, 50% in the lung and/or pleura, 50% in the abdominal viscera, 23% in the central nervous system, and 19% in the skin. Patients younger than 50 years were much more likely to develop metastases in the central nervous system than patients 50 years and older. Seventy-seven (66%) of the 116 patients with metastatic breast cancer received chemotherapy. This proportion decreased with age (p = 0.005), as did the number of schemes per patient. Together, they received 132 chemotherapy schemes, of which 35 (27%) resulted in partial remission or stabilization of the disease process. Ninety-eight patients (84%) received hormonal treatment. This proportion did not differ between the three age groups. Together, they received 216 hormonal treatments, 38 (16%) of which resulted in partial remission or stabilization of the disease process. Seventy-nine patients (68%) received palliative radiotherapy. This proportion decreased with age (p = 0.03). Together, they underwent 216 courses, 176 (77%) of which resulted in relief of the complaints.

**Conclusion:**

Patients aged 70 years and older are less likely to receive chemotherapy or radiotherapy. Part of this difference could be explained by their shorter survival time after the diagnosis of metastatic disease and their lower risk of developing brain and bone metastases. However, more research is needed to understand the age-related differences in the treatment of metastatic breast cancer, and especially how comorbidity and frailty limit therapeutic choices.

## Background

In most western countries the life-time breast cancer risk is around 10%. About 20–40% of these patients will ultimately develop metastatic disease and 4–10% of patients will present with metastatic breast cancer (MBC) at diagnosis [1]. Once metastases are detected, the median survival time is about 24 months, with a range from several months to many years, depending on the site of the metastasis as well as the number of metastatic sites [2-5]. The primary aim of the treatment of a patient who has developed metastatic disease is palliation.

The aims of the current study are to describe and quantify the clinical management of women with metastatic breast cancer, from the time of clinical diagnosis of distant disease until death, and to analyze the response to treatment and overall survival. To determine how clinical management and outcome vary with age, the analyses were performed according to specific age groups.

## Methods

### Data collection

Patients were retrieved from the population-based Eindhoven Cancer Registry (ECR). Since 1989, the ECR has recorded follow-up information on all patients with breast cancer, including the date and site of loco-regional and distant metastasis and the date of death. Information on date of death was obtained from the municipal registries in the area of the Eindhoven Cancer Registry and the Central Bureau for Genealogy. The latter is an institution that collects data on all deceased Dutch citizens via the municipal registries. In this way, information on patients who had moved outside the registry area was also obtained. Patients who died outside the Netherlands were wrongly listed as 'alive'. However, the estimated proportion of these patients was less then 0.3%. All 281 patients with a history of breast cancer who died after December 31, 1999 and had been treated at the Catharina Hospital in Eindhoven or the Máxima Medical Center in Veldhoven were selected for the study. These hospitals are both non-academic, teaching hospitals. According to the data of the ECR, 133 of these 281 patients developed distant metastases, including 11 patients with metastatic disease at the time of diagnosis of the primary tumor. It was assumed that the remaining 148 patients did not die from breast cancer; their mean age at death was 77 years. Five of the 133 patients with metastatic disease had to be excluded, because their medical records could not be traced. Another 12 patients were excluded because their metastases appeared to be related to another primary tumor or were redefined as a locoregional recurrence after review of their medical records. Thus, 116 patients remained available for the study. For each of these 116 patients, detailed information was collected on the site(s) of metastases, diagnostic procedures, treatment, hospitalization and clinical follow-up, starting from the time of diagnosis of distant disease. The first site of metastatic disease and all subsequent sites were recorded separately. For patients receiving systemic treatment, the type of drug and the reason for any postponement or dose reduction were recorded. Response to systemic treatment was measured clinically, biochemically or by radiological imaging. Assessment of treatment response was limited by the retrospective nature of the study. Thus, when evaluating the response to a new endocrine treatment or a new course of chemotherapy, patients with stable disease or partial or complete remission were taken together as one group and considered as responders. In all other cases patients were considered to have progressive disease. According to the information in the medical records, the response to radiotherapy was judged to be positive if the patient reported fewer complaints and negative if the severity of the complaints had not changed or increased. Data on the primary tumor and primary therapies were available from the database of the ECR.

Our study was based on a review of clinical charts only and hence needed no approval from our local ethics committee.

### Statistical analysis

The date of first evidence of metastatic disease was defined as the date of diagnosis. The metastasis-free interval was defined as the interval between the date of diagnosis of the primary tumor and the date of diagnosis of metastastic disease, excluding the patients with metastatic disease at the time of diagnosis of the primary tumor.

To study age-related differences in treatment, response to treatment and survival patients were divided into three age groups: <50 years (n = 34), 50–69 years (n = 62) and 70+ years (n = 20), using the age at initial diagnosis of breast cancer.

The frequency distributions of the different variables between the three age groups were compared by means of the likelihood ratio Chi-square test or, when expected counts were less than five, Fisher's Exact test. The Kruskal Wallis test was used to compare continuous variables between the three age groups. The interval from diagnosis of the primary tumor until diagnosis of metastatic disease and the interval from diagnosis of metastatic disease until death found for the three age groups were compared by the logrank test. The significance level was set at 0.05 for all analyses.

## Results

### Patient and disease characteristics

Patient and disease characteristics are presented in Table 1. Of all 116 patients included in our study, 10 (9%) already had metastatic disease at diagnosis and 106 developed distant disease after a diagnosis of localized breast cancer with or without positive lymph nodes. The mean age of the patients was 58 years (range: 21–88) at the time of diagnosis of the primary tumor and 61.5 years (range: 23–93) at the time of diagnosis of metastatic disease (Figure). No significant differences were observed between age groups as far as the size of the primary tumor, axillary nodal status and the proportion of patients with metastatic disease at the time of diagnosis of the primary tumor were concerned. Also the proportion of patients undergoing surgery, radiotherapy or systemic treatment as part of their primary treatment did not differ between the three age groups.

Among the patients without metastatic disease at diagnosis, the length of the metastasis-free interval tended to be longer for patients 50–69 years than for younger and older patients (p = 0.07).

### Disease progression and survival

Before they died, 70% of the 116 patients developed metastases in the bone, 50% in the lung and/or pleura, 50% in the abdominal viscera, 23% in the central nervous system, and 19% in the skin (Table 1). Patients younger than 50 years were much more likely to develop metastases in the central nervous system than patients 50 years and older. The risk of developing bone or visceral metastases seemed to decrease with age (p = 0.16). The median survival after the diagnosis of metastatic disease was 12 months for patients 70 years and older and 21 months for patients younger than 70 years (p = 0.048).

### Management of metastatic breast cancer

Table 2 shows the differences in the management of the 116 patients with metastatic breast cancer according to age group. As far as diagnostic procedures are concerned, the median numbers of laboratory tests and imaging procedures were significantly lower among patients aged 70 years and older compared to the patients younger than 70 years of age. The proportion of patients visiting a medical oncologist and the number of visits decreased with increasing age. The large majority of the patients was admitted to hospital at least once after the diagnosis of metastatic disease. The median number of admissions was 3, being somewhat higher for patients younger than 50 years than for patients 70 years and older (p = 0.06). The mean duration of admission was significantly longer for patients 50 years and older than for younger patients (p = 0.02). Progression and treatment of metastatic disease were the main reasons for hospital admission in all age groups. Other important reasons for hospital admission were the treatment of co-morbid conditions and the management of treatment-related complications. Of the 116 patients, 53 (46%) died in the hospital.

The proportion of patients with metastatic disease receiving chemotherapy decreased with age (Table 2), as did the number of schemes (Table 3). Patients 20–49 and 50–69 years of age were treated mainly with combination chemotherapy (CAF, CMF or FEC), whereas patients 70 years and older were treated with (less aggressive) monotherapy, such as mitoxantrone and vinorelbine. Treatment delays occurred in about one-third of the cases, mainly due to myelosuppression. Dose reductions were relatively rare and in most cases they were a result of myelosuppression. Thirty-five of the 132 chemotherapy schemes (27%) resulted in partial remission or stabilization of the disease process. Complete remission was observed only once.

The proportion receiving hormonal treatment did not differ between the three age groups (Table 2). Tamoxifen and aromatase inhibitors were usually given as first or second line treatments and megestrol acetate was given to one-third of the patients, mainly as a third-line drug (Table 4). Thirty-eight of the 216 hormonal treatments (16%) resulted in partial remission or stabilization of the disease process.

Of the 116 patients, 79 received palliative radiotherapy. The proportion decreased with age (Table 2). Together, the 79 patients underwent 216 courses, of which 160 (70%) were less than 20 Gy and 176 (77%) resulted in a relief of the complaints of the patient (Table 5).

## Discussion

The current study gives an accurate picture of the clinical management of women with metastatic breast cancer in two general hospitals in the Netherlands. The care for these patients is characterized by extensive laboratory testing and diagnostic imaging and by intensive treatment, associated with regular hospital admission. Furthermore, frequent outpatient visits to the medical oncologist, surgeon, radiation oncologist and other disciplines involved in the treatment of metastatic disease were required.

Substantial differences were observed between younger and older patients with metastatic breast cancer as far as their prognosis, the metastatic pattern and the management of their disease are concerned. As has been confirmed by other studies [2,6,7], elderly patients had a worse prognosis than their younger counterparts. They also had a lower risk of developing bone, visceral and brain metastases during the course of their disease, which is in line with other studies [8-10]. One hypothesis, originating from studies among patients receiving paclitaxel and trastuzumab, is that the central nervous system is an important sanctuary site and that the higher risk of brain metastases among younger patients is associated with better systemic control of extracerebral metastases and a prolonged survival [11,12].

Patients aged 70 years and older were less likely to receive chemotherapy or radiotherapy and underwent fewer staging procedures. Part of this difference can be explained by their shorter survival time after diagnosis of metastases and the fact that metastases to the bone and brain occurred less frequently. A further explanation for the decrease in the number of staging procedures with increasing age is that the therapeutic consequences become less relevant, especially in the case of (serious) co-morbidity, which is present in about two-thirds of the patients of 75 years and older [13].

As was confirmed by our findings, planning therapy is not always straightforward for older patients because they are more likely to present with co-morbidity and frailty which limits the therapeutic choices [14]. Although only a few elderly patients received chemotherapy in our study, it was well-tolerated with no need for dose reductions. Christman and colleagues have found that elderly women in overall good health are able to tolerate chemotherapy as well as their younger counterparts [15]. However, there is some evidence that although older patients do not differ from their younger counterparts in their acceptance of chemotherapy, they are less willing to trade survival for current quality of life [16]. For the decision on the administration of chemotherapy to elderly patients, it is important to identify the variables that influence their tolerance to this treatment. Extermann and colleagues have attempted to design a predictive risk score for elderly cancer patients, including patient-related and chemotherapy-related variables that correlate independently with toxicity [17]. Although the results of their pilot study should be interpreted with care, diastolic blood pressure and bone marrow invasion were found to be associated with toxicity, in addition to the known toxicity of the different chemotherapeutic regimens. As for adjuvant chemotherapy, a comprehensive geriatric assessment may be a useful tool to predict the risk of toxicity and/or the efficacy of systemic treatment for elderly patients [18].

Our study has several limitations. First, when considering the results it should be realized that the number of patients was small and that the large number of tests for possible associations carries the added risk that apparently significant differences will occur by chance alone. Second, our study is based on patients treated in two non-academic, teaching hospitals and it is not sure that comparable results would have been obtained for patients treated in an academic centers or in smaller non-teaching hospitals. And third, the lack of information on hormone receptor status is limiting the reconstruction of the decision-making process on the use of endocrine treatment.

It was not the aim of our study to quantify the costs associated with the treatment of patients with metastatic breast cancer. In a Canadian study of 75 patients the mean total health care costs during the interval from diagnosis of first recurrence or metastasis until death were estimated at 36,474 Canadian Dollars in 1995 (25,686 Euros), including homecare. Inpatients costs accounted for more than 50% of the total costs in all age groups [19]. Another study demonstrated the dependency of the costs on the duration and the site of metastatic involvement [20]. In 1988, Holli and Hakama questioned the cost-effectiveness of the treatment of metastatic breast cancer and suggested that resources for diagnostic investigations and treatment of this group could better be used to improve quality of life and to conserve resources [21]. This opinion was based on their observation that response is generally poor and only rarely translates into detectable survival advantages. However, for metastatic breast cancer, treatment can be recommended even in the absence of survival improvement, if it contributes to symptom control and, consequently, the quality of life. Cost-utility analyses are recommended to solve this issue [22]. At the same time, a greater effort is needed to develop reliable predictors of response to treatment for individual patients with metastatic breast cancer in an effort to improve quality of life and manage costs [23].

## Conclusion

The treatment of patients with metastatic breast cancer is changing rapidly and it is very likely that the agents used in our study no longer represent current treatment. For example, taxanes have rapidly become first-line therapy and many new drugs, such as trastuzumab, have become available; furthermore multiple agent therapy has been given a more prominent place [24]. Although the agents have altered, it is not likely that changes have taken place in the considerations to start second or third line chemotherapy or endocrine treatment, and therefore the results of the current retrospective study may serve as a reference for the implementation and monitoring of new diagnostic and therapeutic interventions and their cost-effectiveness. However, more research is needed to understand the age-related differences in the treatment of metastatic breast cancer, and especially how co-morbidity and frailty limit therapeutic choices. It should be realized that despite the broadening of the therapeutic spectrum, quality of life and symptom palliation remain the cornerstones of decision-making for women of all age groups with metastatic breast cancer.

## Competing interests

The author(s) declare that they have no competing interests.

## Authors' contributions

KM and LVPF performed the data analysis and quality assessment. KM was responsible for collecting the data and the data entry. KM and AV were the principal authors. LVPF and AV were the principal investigators. GJC, GV and MJCS have made substantial contributions to the conception and design of the study and the interpretation of the data. GJC, GV, GAPN, RMHR and MJCS have been involved in critically revising the manuscript and approval of the final version.

## Pre-publication history

The pre-publication history for this paper can be accessed here:



## Figures and Tables

**Figure 1:**
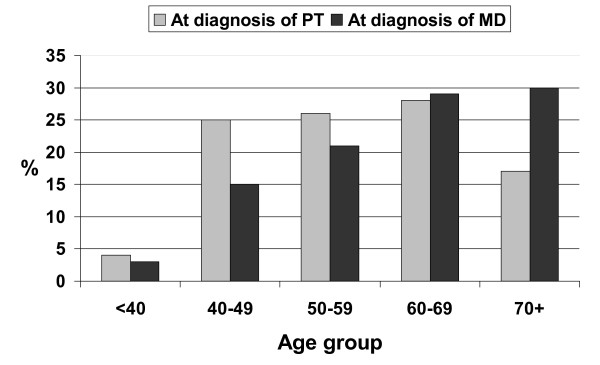
Age distribution at time of diagnosis of primary tumor (PT) and at the time of diagnosis of metastatic disease (MD) (n = 116).

**Table 1:** Characteristics of primary tumor and metastatic disease according to age (%)

Characteristic	Age at time of diagnosis of primary tumor						
		
	20–49 (n = 34)		50–69 (n = 62)		70+ (n = 20)		p-value
				
	No.	(%)	No.	(%)	No.	(%)	
Tumor size							
T1	15	(44)	27	(43)	7	(35)	0.60
T2	16	(47)	25	(40)	7	(35)	
T3	0	(0)	1	(2)	0	(0)	
T4	3	(8)	8	(12)	6	(30)	
Tx	0	(0)	1	(2)	0	(0)	
Lymph node status							
N+	19	(55)	32	(51)	8	(40)	0.65
N-	11	(32)	21	(33)	6	(30)	
Unknown	4	(11)	9	(14)	6	(30)	
Metastasis							
M0	31	(91)	55	(88)	19	(95)	0.91
M1	3	(8)	7	(11)	1	(5)	
Histology							
ductal	21	(61)	35	(56)	14	(70)	0.89
lobular or mixed	9	(25)	19	(29)	3	(20)	
other	4	(11)	8	(12)	3	(10)	
Primary therapy							
surgery	33	(97)	59	(95)	19	(95)	1.00
radiotherapy	25	(73)	42	(67)	14	(70)	0.86
systemic therapy	21	(61)	36	(58)	14	(70)	0.64
other	0	(0)	1	(1)	0	(0)	-
*Metastatic disease*							
Interval (months) from diagnosis primary tumor until diagnosis of metastatic disease							
Median (range)	25 (14–47)		42 (17–73)		23 (14–48.5)		0.07
Interval from diagnosis of metastatic disease until death							
Median (range)	21 (11–45)		22 (9–50)		12 (6–31.5)		0.048
First site of metastatic disease							
bone	14	(41)	18	(29)	5	(25)	0.21
lung and/or pleura	9	(26)	20	(32)	8	(40)	
abdominal viscera	3	(8)	12	(19)	1	(5)	
other	8	(23)	12	(19)	6	(30)	
All metastatic sites (until death)							
bone	27	(79)	43	(69)	11	(55)	0.16
lung and/or pleura	16	(47)	31	(50)	11	(55)	0.32
abdominal viscera	20	(58)	33	(53)	5	(25)	0.16
central nervous system	15	(44)	11	(17)	1	(5)	0.001
skin	6	(18)	12	(19)	5	(25)	0.80
other	9	(26)	14	(23)	4	(20)	0.88
Co-morbid conditions							
Previous cancer	1	(3)	2	(3)	2	(10)	0.46
Lung disease*	5	(15)	8	(13)	5	(25)	0.45
Cardiovascular disease**	1	(3)	23	(37)	12	(60)	<0.0001
Diabetes	0	(0)	5	(8)	7	(35)	0.0002

* Asthma/COPD, pulmonary fibrosis

** Intermittent claudication, Myocardial infarction, CABG, Angina, Heart failure, CVA, TIA, Hypertension

**Table 2:** Management of patients with metastatic breast cancer, according to age group

	age at time of diagnosis of primary tumor			
		
	20–49 (n = 34)	50–69 (n = 62)	70+ (n = 20)	p-value
*Diagnostic procedures*				
Laboratory tests:				
No. of patients	34 (100%)	61 (98%)	18 (90%)	0.06
No. of tests per patient*	45 (26–68)	35 (20–58)	13 (8–33)	0.005
Imaging:				
No. of patients	33 (97%)	62 (100%)	19 (95%)	0.09
No. of exams per patient*	17 (13–32)	16.5 (9–26)	7 (4–19)	0.009
Histological or cytological confirmation:				
No. of patients	20 (58%)	37 (59%)	10 (50%)	0.69
*Hospital admission*				
No. of patients	31 (91%)	56 (90%)	15 (75%)	0.15
No. of admissions (median) *	4 (2–5)	3 (2–4)	2 (1–4)	0.06
Mean duration per admission (days) *	8.5 (5–10)	12 (6.5–17)	12 (4.6–16)	0.02
Reason for hospital admission:				
Treatment (surgery, radiotherapy, systemic)	22 (65%)	35 (56%)	3 (15%)	0.001
Treatment-related complications	10 (29%)	9 (15%)	1 (5%)	0.05
Disease progression	30 (88%)	49 (79%)	14 (70%)	0.25
Disease progression and treatment-related complications	8 (24%)	15 (24%)	3 (15%)	0.68
Not related to breast cancer	0 (0%)	11 (18%)	3 (15%)	0.03
*Visits to medical oncologist*				
No. of patients (%)	34 (100%)	61 (98%)	17 (85%)	0.007
No of visits per patient*	25 (14–42)	19 (8–32)	9 (7–18)	0.02
*Treatment*				
Chemotherapy:				
No. of patients	27 (79%)	35 (56%)	5 (25%)	0.005
Hormonal treatment:				
No. of patients	28 (82%)	54 (87%)	16 (80%)	0.68
Radiotherapy:				
No. of patients	27 (79%)	43 (69%)	9 (45%)	0.03

* median with 25% and 75% quartiles between parentheses

**Table 3:** Palliative chemotherapy, according to age group

Chemotherapy	Age at time of diagnosis of primary tumor			
	
	20–49 (n = 27)	50–69 (n = 35)	70+ (n = 5)	total (n = 67)
Number of schemes				
1	8	14	3	25
2	11	14	2	27
3	4	4	0	8
4	4	2	0	6
5	0	1	0	1
Total number of schemes	58	67	7	132
Drugs used				
CAF	16	15	0	31
CMF	14	16	0	30
FEC	7	3	0	10
taxotere	9	7	0	16
mitoxantrone	1	8	3	12
vinorelbine	4	5	3	12
other	7	13	1	21
Reason for postponement				
bone marrow suppression	4	11	1	16
fever	2	0	0	2
toxicity	2	6	0	8
infection	1	2	2	5
Doses				
100%	52	57	7	116
80%	1	2	0	3
75%	3	5	0	8
70%	0	1	0	1
50%	1	2	0	3
Reason for dose reduction				
bone marrow suppression	5	6	0	11
toxicity	1	2	0	3
other	1	3	0	4
Response				
yes*	15	20	0	35
no (progressive disease)	43	46	5	91

*stable disease, partial or complete remission

**Table 4:** Palliative hormone therapy, according to age group

Hormonal treatment	Age at time of diagnosis of primary tumor			
	
	20–49 (n = 28)	50–69 (n = 54)	70+ (n = 16)	total (n = 98)
Total number of courses	70	112	34	216
Drug				
tamoxifen	27	35	8	70
aromatase inhibitor	21	40	11	72
megestrol acetate	13	24	8	45
other	9	13	7	29
Response				
yes*	7	28	3	38
no (progressive disease)	63	82	31	176

*stable disease, partial or complete remission

**Table 5:** Palliative radiotherapy, according to age group

Radiotherapy	Age at time of diagnosis of primary tumor			
	
	20–49 (n = 27)	50–69 (n = 43)	70+ (n = 9)	total (n = 79)
Total number of courses	94	113	20	227
Dose intensity (Gy)				
0–9	37	38	10	85
10–19	29	38	8	75
20–29	24	25	0	49
30+	4	12	2	18
Response*				
positive effect	72	89	15	176
no effect	20	24	5	49

*positive effect: patient reporting less complaints; no effect: patients reporting no change or increased severity of complaints

## References

[B1] Edwards MJ, Gamel JW, Feuer EJ (1998). Improvement in the prognosis of breast cancer from 1965 to 1984. J Clin Oncol.

[B2] Chang J, Clark GM, Allred DC, Mohsin S, Chamness G, Elledge RM (2003). Survival of patients with metastatic breast carcinoma: importance of prognostic markers of the primary tumor. Cancer.

[B3] Andre F, Slimane K, Bachelot T, Dunant A, Namer M, Barrelier A, Kabbaj O, Spano JP, Marsiglia H, Rouzier R, Delaloge S, Spielmann M (2004). Breast cancer with synchronous metastases: trends in survival during a 14-year period. J Clin Oncol.

[B4] Insa A, Lluch A, Prosper F, Marugan I, Martinez-Agullo A, Garcia-Conde J (1999). Prognostic factors predicting survival from first recurrence in patients with metastatic breast cancer: analysis of 439 patients. Breast Cancer Res Treat.

[B5] Solomayer EF, Diel IJ, Meyberg GC, Gollan C, Bastert G (2000). Metastatic breast cancer: clinical course, prognosis and therapy related to the first site of metastasis. Breast Cancer Res Treat.

[B6] Coleman RE, Smith P, Rubens RD (1998). Clinical course and prognostic factors following bone recurrence from breast cancer. Br J Cancer.

[B7] Ryberg M, Nielsen D, Osterlind K, Skovsgaard T, Dombernowsky P (2001). Prognostic factors and long-term survival in 585 patients with metastatic breast cancer treated with epirubicin-based chemotherapy. Ann Oncol.

[B8] Lai R, Dang CT, Malkin MG, Abrey LE (2004). The risk of central nervous system metastases after trastuzumab therapy in patients with breast carcinoma. Cancer.

[B9] Schouten LJ, Rutten J, Huveneers HA, Twijnstra A (2002). Incidence of brain metastases in a cohort of patients with carcinoma of the breast, colon, kidney, and lung and melanoma. Cancer.

[B10] Evans AJ, James JJ, Cornford EJ, Chan SY, Burrell HC, Pinder SE, Gutteridge E, Robertson JF, Hornbuckle J, Cheung KL (2004). Brain metastases from breast cancer: identification of a high-risk group. Clin Oncol (R Coll Radiol).

[B11] Freilich RJ, Seidman AD, DeAngelis LM (1995). Central nervous system progression of metastatic breast cancer in patients treated with paclitaxel. Cancer.

[B12] Duchnowska R, Szczylik C (2005). Central nervous system metastases in breast cancer patients administered trastuzumab. Cancer Treat Rev.

[B13] Coebergh JW, Janssen-Heijnen ML, Post PN, Razenberg PP (1999). Serious co-morbidity among unselected cancer patients newly diagnosed in the southeastern part of The Netherlands in 1993-1996. J Clin Epidemiol.

[B14] Kimmick G, Muss HB (2004). Breast cancer in older patients. Semin Oncol.

[B15] Christman K, Muss HB, Case LD, Stanley V (1992). Chemotherapy of metastatic breast cancer in the elderly. The Piedmont Oncology Association experience [see comment]. Jama.

[B16] Yellen SB, Cella DF, Leslie WT (1994). Age and clinical decision making in oncology patients. J Natl Cancer Inst.

[B17] Extermann M, Chen H, Cantor AB, Corcoran MB, Meyer J, Grendys E, Cavanaugh D, Antonek S, Camarata A, Haley WE, Balducci L (2002). Predictors of tolerance to chemotherapy in older cancer patients: a prospective pilot study. Eur J Cancer.

[B18] Biganzoli L, Goldhirsch A, Straehle C, Castiglione-Gertsch M, Therasse P, Aapro M, Minisini A, Piccart MJ (2004). Adjuvant chemotherapy in elderly patients with breast cancer: a survey of the Breast International Group (BIG). Ann Oncol.

[B19] Wai ES, Trevisan CH, Taylor SCM, Mates D, Jackson JS, Olivotto IA (2001). Health system costs of metastatic breast cancer. Breast Cancer Res Treat.

[B20] Hurley SF, Huggins RM, Snyder RD, Bishop JF (1992). The cost of breast cancer recurrences. Br J Cancer.

[B21] Holli K, Hakama M (1989). Treatment of the terminal stages of breast cancer. Bmj.

[B22] (1996). Outcomes of cancer treatment for technology assessment and cancer treatment guidelines. American Society of Clinical Oncology. J Clin Oncol.

[B23] Robain M, Pierga JY, Jouve M, Asselain B, Dieras V, Beuzeboc P, Palangie T, Dorval T, Extra JM, Scholl S, Pouillart P (2000). Predictive factors of response to first-line chemotherapy in 1426 women with metastatic breast cancer. Eur J Cancer.

[B24] Cardoso F, Di LA, Lohrisch C, Bernard C, Ferreira F, Piccart MJ (2002). Second and subsequent lines of chemotherapy for metastatic breast cancer: what did we learn in the last two decades?. Ann Oncol.

